# Forest restoration, biodiversity and ecosystem functioning

**DOI:** 10.1186/1472-6785-11-29

**Published:** 2011-11-24

**Authors:** Raf Aerts, Olivier Honnay

**Affiliations:** 1 Division Forest, Nature and Landscape, University of Leuven, Celestijnenlaan 200E-2411, BE-3001 Leuven, Belgium; 2 Laboratory of Plant Ecology, University of Leuven, Kasteelpark Arenberg 31-2435, BE-3001 Leuven, Belgium

## Abstract

Globally, forests cover nearly one third of the land area and they contain over 80% of terrestrial biodiversity. Both the extent and quality of forest habitat continue to decrease and the associated loss of biodiversity jeopardizes forest ecosystem functioning and the ability of forests to provide ecosystem services. In the light of the increasing population pressure, it is of major importance not only to conserve, but also to restore forest ecosystems.

Ecological restoration has recently started to adopt insights from the biodiversity-ecosystem functioning (BEF) perspective. Central is the focus on restoring the relation between biodiversity and ecosystem functioning. Here we provide an overview of important considerations related to forest restoration that can be inferred from this BEF-perspective.

Restoring *multiple *forest functions requires multiple species. It is highly unlikely that species-poor plantations, which may be optimal for above-ground biomass production, will outperform species diverse assemblages for a combination of functions, including overall carbon storage and control over water and nutrient flows. Restoring *stable *forest functions also requires multiple species. In particular in the light of global climatic change scenarios, which predict more frequent extreme disturbances and climatic events, it is important to incorporate insights from the relation between biodiversity and stability of ecosystem functioning into forest restoration projects. Rather than focussing on species per se, focussing on *functional diversity *of tree species assemblages seems appropriate when selecting tree species for restoration. Finally, also *plant genetic diversity *and *above - below-ground linkages *should be considered during the restoration process, as these likely have prominent but until now poorly understood effects at the level of the ecosystem.

The BEF-approach provides a useful framework to evaluate forest restoration in an ecosystem functioning context, but it also highlights that much remains to be understood, especially regarding the relation between forest functioning on the one side and genetic diversity and above-ground-below-ground species associations on the other. The strong emphasis of the BEF-approach on functional rather than taxonomic diversity may also be the beginning of a paradigm shift in restoration ecology, increasing the tolerance towards allochthonous species.

## Review

Globally, forests cover nearly one third of the land area and contain over 80% of terrestrial biodiversity [1]. The income of more than 1.6 billion people depends on forests and sustainable management of forests can contribute to sustainable development, poverty eradication and the achievement of internationally agreed development goals [1,2]. Despite increasing efforts for sustainable forest management and forest conservation [3], the extent of forest habitat, in particular in the tropics, continues to decrease, mainly by forest conversion to agriculture and land uses related to urban population growth [4,5]. Between 1980 and 2000 more than half of the new agricultural land across the tropics was obtained by clearing intact forests [6,7]. Also, many disturbed and secondary forests, which are increasingly important habitat for many forest species [8,9], are eventually cleared for agricultural purposes.

In the remaining forests and forest fragments, decreasing habitat patch sizes result in increased deleterious edge effects [10] and decreasing plant and animal population sizes [11], which, in turn, may lower population viability and genetic variation [12,13]. The negative effects of forest fragmentation and isolation are expected to be exacerbated by other anthropogenic threats such as fire [14,15], in particular in the light of global climatic change [16,17]. Parallel to forest loss and forest fragmentation, cryptic deforestation [18,19] - the selective logging and internal degradation of forests - alters forest structure and plant communities, jeopardizing biodiversity, regeneration capacity and vitality of forests [20]. The simultaneous reduction of both forest quantity and quality is expected to lead to massive extinction of many species inhabiting forest habitats [21]. For a wide range of taxa, including trees and lianas, birds, fruit-feeding butterflies, leaf-litter amphibians, large mammals, epigeic arachnids, lizards, dung beetles and bats, biodiversity has been shown to decline significantly over a forest degradation gradient, from primary over secondary to plantation forest [22].

Loss of forest biodiversity may seriously jeopardize the functioning of forest ecosystems (*i.e*. the activities, processes or properties of forests, such as decomposition of organic matter, soil nutrient cycling and water retention), and consequently the ability of forest to provide ecosystem services [23]. Ecosystem services have been defined as the benefits that people obtain from ecosystems [24] and have been categorized into four broad categories. These include *provisioning services *such as food, water, timber, and fiber; *regulating services *that affect climate (e.g. though carbon sequestration), pollination, biological pest control, floods, disease, wastes, and water quality; *cultural services *that provide recreational, aesthetic, and spiritual benefits; and *supporting services *such as soil formation, photosynthesis, and nutrient cycling [25-27].

Clearly, the role of forests as sanctuaries of biodiversity and as providers of ecosystem services cannot be overestimated. In the light of the increasing human population, however, conserving the remaining forests and their biodiversity, functions and services of forests is unlikely to be sufficient [7]. To meet the increasing demands for ecosystem services provided by forests - in particular the many provisioning services of forests as many people heavily rely on forests for livelihoods and products such as timber, medicines, thatch, fiber and meat [1] - large-scale (passive or active) forest restoration is probably the only solution that will be effective in the long term [28-30]. Establishing short-rotation single- or multiple-species plantations on degraded soils, restoration plantings in secondary forests or assisted regeneration in selectively logged forest are a few examples of the wide spectrum of forest restoration approaches [31]. They all have in common that they consist of management interventions that aim at recovering ecosystems that have been degraded, damaged or destroyed by human activities [29,32]. Ecological restoration is therefore an important practice that may increase levels of biodiversity in human-altered ecosystems [33] and may mitigate the impact of climate change [34]. To this end, restoration ecology has adopted insights from both community and ecosystem ecology, and more recently, from the integrated biodiversity-ecosystem functioning (BEF) perspective [35-37]. The main aim of this article is to discuss how forest restoration may benefit from insights originating from the emerging BEF framework.

## Traditional approaches to ecological restoration

### The community approach

**Figure 1:**
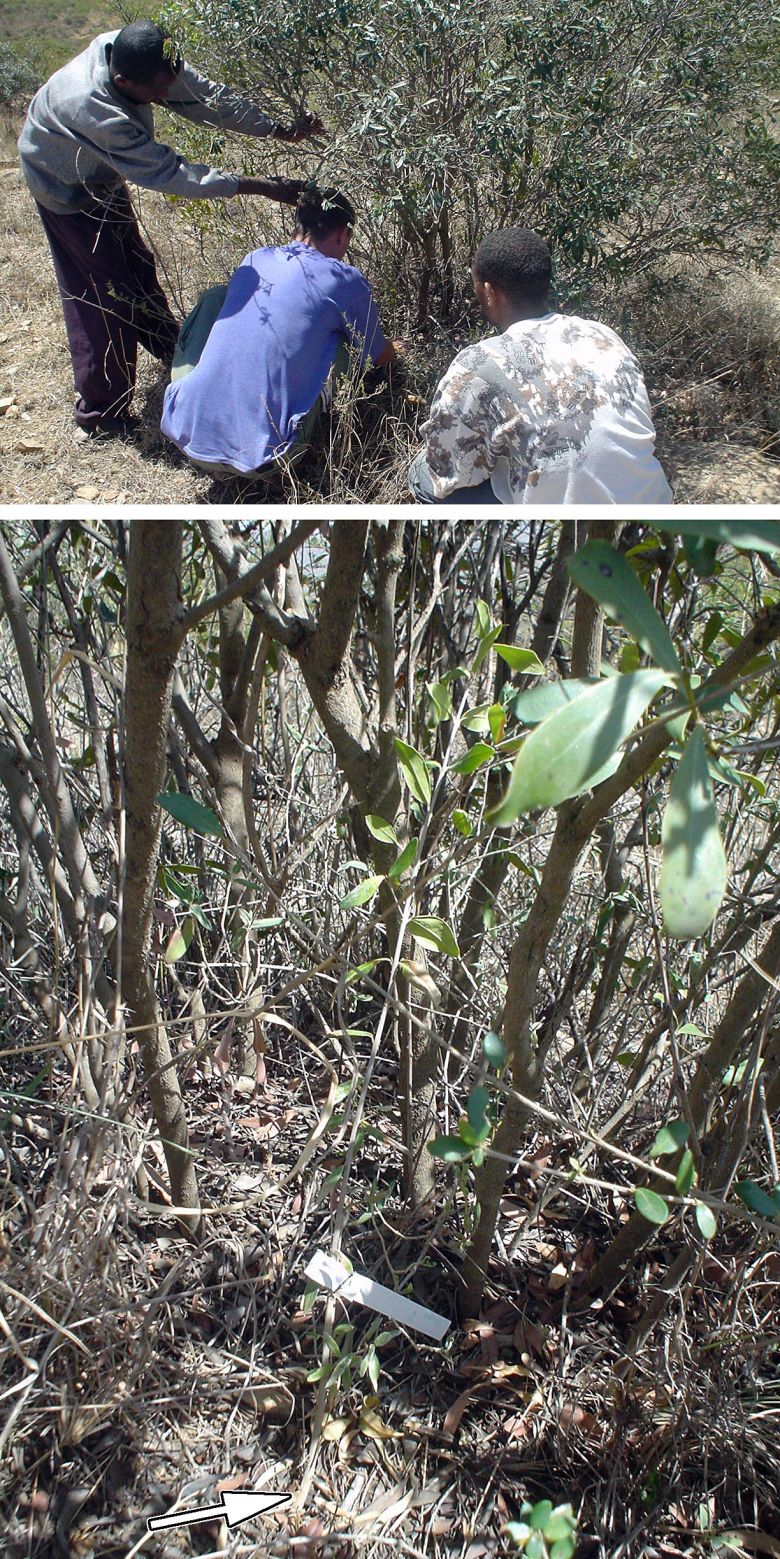
**Seedling planting and ecological forest restoration**. Planting late-successional tree species under early-successional shrubs can be an effective means of restoring forests under high abiotic stress. Tree planting under facilitating *nurse shrubs *is a typical example of the community approach to forest restoration. This figure shows the planting of an African wild olive seedling (*Olea europaea *ssp. *cuspidata*) under the canopy of *Euclea racemosa *rather than in the open space between the present shrubs. See [43] for details. Tsegaye Gebremariam, Raf Aerts and Bisrat Haile agreed to be photographed in the field.

A biological community is a group of organisms that interact and share an environment. Within a community, organisms may compete for the same resources (competition), profit from the presence of other organisms (facilitation) [38,39] or use other organisms as a food source (trophic interaction) [35]. In stable communities, these interactions lead to predictable, directional changes in community structure known as ecological succession. Succession is an important guiding principle in the community approach to ecological restoration [40]. The restoring forest is a dynamic ecosystem, with changing species composition and forest structure, but interventions and management steer the forest towards a desired climax or pre-disturbance community structure. These interventions are usually designed to accelerate natural succession or to bypass intermediate successional phases. Basically, the community approach is focussing on restoring forest biodiversity per se. The many studies that apply facilitation as a restoration tool of woody communities [41] are typical examples of the community approach to forest restoration. Planting late-successional tree species (protégé species) under early-successional shrubs (nurse species) has been shown to be an effective means of restoring forests under high abiotic stress [42,43] (Figure 1).

Insights from alternative stable states theory have also been useful to guide restoration practices that focus on community structure [44]. In severely degraded systems, alternative stable states may make efforts to restore pre-disturbance communities difficult, if not impossible [45]. In such cases, a single intervention may not suffice to induce forest regrowth: succession fails and the community is blocked in a low diversity/low biomass state. Exclusion of grazing animals may be an effective means for woodland restoration in degraded drylands, but only when soil moisture conditions also improve. Wet pulses caused by climatic oscillations such as the El Niño Southern Oscillation may provide such necessary additional impulse to induce a regime shift that leads to forest restoration [46]. Similarly, planting and sowing of late successional tree species (an intervention to overcome seed limitation) has been found effective for the restoration of highly complex forest on bauxite mined sites, but only after careful site preparation and topsoil handling or replacement (interventions to overcome survival limitation caused by soil compaction, decreased soil porosity and infiltration capacity, and the loss of soil biota) [47,48].

### The ecosystem approach

Restoration of species richness and community structure over time implies increasing ecosystem complexity and functionality [40]. In the ecosystem approach, restoration of ecosystem functions such as primary production, energy flows and nutrient cycles, is the guiding principle on which restoration efforts are based [35]. Basically, this approach aims at restoring suitable abiotic conditions that allow (passive) recolonization of species. The ecosystem perspective typically starts from a landscape point of view, building on spatial heterogeneity and broad spatial scales [49]. The connections or barriers between neighbouring ecosystems have an effect on the resource balances and set limits on the communities that can be restored [50]. Reforestation of degraded sites with trees that alter the physical and chemical characteristics of the soil and that affect the biochemical cycles through litter fall or root activity presents a typical example of the ecosystem approach to forest restoration [51,52].

## The biodiversity - ecosystem function approach to ecological forest restoration

The study of the relation between biodiversity and ecosystem functioning is a rapidly growing field (see the volume edited by Naeem *et al*. [53] for an exhaustive state of the art). The traditional view that has dominated ecology until the 1990's started from the idea that species distribution patterns resulted directly from the abiotic and biotic (species interactions) components determining the environment. In the early 1990's, however, this view was challenged, when one started to realize that species diversity also affects the abiotic environment, and even the functioning of ecosystems [54]. The functioning of an ecosystem incorporates processes such as decomposition of organic matter, fixation of carbon, nutrient and water cycling and degradation of toxic compounds. Meta-analyses of the results of mainly small-scale biodiversity experiments have shown that, on average, ecosystem functions increase with increasing species number [e.g. [55]]. The success of the idea that biodiversity affects ecosystem properties and functions - some have called it a paradigm shift in ecology [56] - can be explained by the fact that it offers a comprehensive framework to evaluate the consequences of biodiversity loss caused by human activities, and at the same time provides a powerful incentive for biodiversity conservation and ecological restoration [37,57].

**Figure 2:**
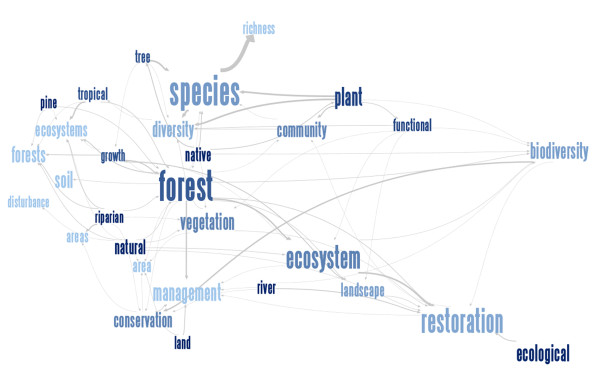
**Related concepts in the scientific literature on forest restoration, biodiversity and ecosystem functioning**. The relationship between the most widely used words (30 of 2745 terms) in the abstracts of scientific literature on forest restoration, biodiversity and ecosystem functioning (BEF). Data were obtained from Thomson Reuters Web of Science using the query Topic = (biodiversity ecosystem function*) refined by Topic = (restoration) AND Topic = (forest*). The diagram shows that, even in the BEF literature, *functional (bio)diversity *has received less attention than *species richness *and *(plant) species diversity *thus far (an interactive online version shows the number of occurrences for each word and word pair and the contexts of each word pair and is available at http://www-958.ibm.com/v/116799).

Naeem [35] was the first to propose that restoration ecology may benefit from insights from the BEF framework, and this idea has been further elaborated by Wright *et al*. [36]. Here we build on these ideas and put them in a forest restoration context. In contrast to more traditional approaches, restoration based on the BEF perspective strongly focuses on restoring the relationship between biodiversity and ecosystem functioning [35]. In what follows we list some important considerations regarding forest restoration that can be inferred from the BEF framework. We are aware that foresters have already adopted the BEF framework in setting up large experiments where the effects of tree species richness on ecosystem functions are evaluated [e.g., [58,59]]. Nevertheless, we believe that forest restoration efforts may benefit from such an overview, in particular since ecosystem functioning and functional (bio)diversity has received very little attention in a forest restoration context so far (Figure 2).

### Restoring multiple forest functions requires multiple species

One of the major functions of forest ecosystems is carbon fixation [60], which is directly related to the ecosystem services carbon sequestration and the provision of fire and construction wood. There is evidence that tree diversity has a positive effect on ecosystem production (see Thompson *et al*. [61] for an overview). Based on the largest data set ever analysed in this context to date (12.000 permanent forest plots in eastern Canada), Paquette & Messier [62] reported that, after controlling for environmental and climate differences between plots, tree productivity was positively related to stand biodiversity. These results confirm earlier work in 5000 permanent plots in Mediterranean forests across Catalonia (NE Spain) [63]. In a reforestation context, Piotto *et al*. [64] found that mixed plantations in Costa Rica performed better than monocultures for all growth variables considered, including height, diameter at breast height, volume, and above-ground biomass. Also in natural stands of tropical forest with high environmental and spatial variation, positive effects of tree species diversity on tree carbon storage were found [65]. Positive effects of tree diversity on above-ground productivity are certainly not an universal pattern, however [61,66], and above-ground biomass production and soil carbon fixation may also respond differently to tree diversity in plantation forests [67]. This corroborates the result of a meta-analysis of BEF experiments where it was found that high biodiversity treatments do not always outperform the best performing monoculture [55]. In a forest restoration context, where fast growing tree species with strong global timber markets are readily available, this may suggest that monocultures are an option. However, evidence is accumulating that focussing on one single ecosystem function often overlooks an important aspect of biodiversity: the possibility of one species to contribute to different ecosystem functions at the same time [68]. Because different species often influence different ecosystem functions, focussing on one function in isolation will strongly underestimate the biodiversity required for maintaining an ecosystem with multiple functions, at multiple times and places in a changing environment [69]. Although the evidence only comes from grasslands and aquatic environments so far, it convincingly shows that species redundancy is unlikely to occur when several ecosystem functions and services are considered in combination [68-71].

Therefore, it is highly unlikely that species poor plantations will outperform species diverse tree assemblages for a combination of forest ecosystem functions [28], including above-ground biomass production, disease resistance, carbon fixation, nectar provision, erosion control, water capitation, N_2_-fixation and fruit production. It is therefore of special importance that reforestation efforts clearly define the ecosystem services and functions that the restored forest is intended to deliver. Also, it is important to realize that ecosystem functions of restoring forests may change over time because of changes in tree sizes, forest structure and relative importance of functional groups, even if there are no changes in tree species composition [72]. Finally, it should be noticed that although there is already some knowledge on the effects of tree diversity on forest productivity, it is not known how understory shrub diversity, and even herbaceous species, affect forest productivity or other ecosystem functions. This may, for example, happen through these species' impacts on litter decomposition, on water capture and on the diversity of soil biota [73].

### Restoring stable forest functions requires multiple species

The hypothesis that larger species diversity leads to higher stability of ecosystem functioning has been a point of debate for half a century, and it has re-emerged within the BEF framework [53,74,75]. The main ideas behind the biodiversity *vs*. ecosystem stability concept are functional response diversity and functional compensation [61,76]. This occurs when positive changes in the level of functioning of one species (a species becoming functionally dominant) are associated with negative changes in the functioning of other species. This compensation drives the stabilization of ecosystem properties such as biomass production [77]. Basically, the stability of the functioning of an ecosystem can be measured in three ways: i) the long term variability of an ecosystem property through time in relation to background environmental variation (*variance*); ii) the impact (*resistance*); and iii) the recovery (*resilience*) of ecosystem properties to discrete disturbances [61,78]. As it is expected that these discrete and extreme disturbances such as extreme climate events and pest and disease outbreaks will become more frequent under the predicted climate change [79], it is very important to incorporate insights from the relation between biodiversity and stability of ecosystem functioning into forest restoration projects. It is crucial to realize that, just as the degree of species redundancy decreases when multiple ecosystem functions are considered (see earlier), there is currently strong experimental evidence that in changing environments, more species are required to guarantee ecosystem functioning than in constant environments [e.g., [69,80]].

Evidence for the latter comes from studies that have related forest tree diversity with measures of stability of forest ecosystem functioning. Lloret *et al*. [81] used satellite imagery to estimate the impact of the extreme 2003 summer drought on canopy greenness of different forests types in Spain, by quantifying the NDVI (normalized difference vegetation index). NDVI correlates with ecosystem CO_2 _fluxes. These authors reported a positive relation between woody species diversity and resistance of canopy greenness against drought in forests on dry locations, whereas no such relation was discovered in more moist forests. Similarly, DeClerck *et al*. [82] related stability in stand productivity across 64 years with conifer diversity in the Sierra Nevada, USA. They found a significant relation between species richness and resilience of stand productivity after recurrent severe droughts. Resistance to drought was, however, not related to species diversity. These studies partly support positive biodiversity effects on stability of biomass production, but they also show that patterns may be complex, vary across ecosystem types, and depend on the measures that are used to quantify stability. In any case, temporal stability of ecosystem functioning is an important consideration for projects aiming at forest restoration, especially under the current global change scenario. Again, it is not known whether understory shrubs and herbaceous species contribute to the stability of forest ecosystem functioning.

### Focus on functional diversity rather than on taxonomic diversity

Whereas general biodiversity measures are based on taxonomy in the first place (species presence or absence), functional diversity measures relate to what organisms effectively do in an ecosystem, quantify the distribution of traits in a community or measure the relative magnitude of species similarities and differences. How to best measure functional diversity is a much debated question, but Cadotte *et al*. [83] summarize five useful multivariate functional diversity measures. Some authors have suggested that functional diversity measures are particularly suitable or even better to predict the interactions between biodiversity and ecosystem processes [83-85]. Using a tree diversity index based on among-species variation in seed mass, wood density and maximum height Paquet and Messier [62] showed that this measure outperformed a taxonomically based diversity index in explaining tree productivity. Bunker *et al*. [86] demonstrated that removing certain functional groups from a tropical forest had more important effects on the above-ground carbon pool than randomly removing species. Vila *et al*. [63], on the contrary, reported that functional group richness performed worse than tree species richness, but this was likely due to a rather rudimentary functional group delineation. Thus, when selecting tree species for forest restoration, these findings suggest focusing on functional groups based on relevant plant traits. While these traits are readily available for species from temperate regions by now, the establishment of plant trait databases for tropical tree species and the centralisation of all available data in a general database are important works in progress [87,88]. Maximizing functional diversity can be achieved by quantifying the functional diversity of the species mix used for restoration. This can be done by delineating emergent or functional groups (assemblages of species performing similar functional roles) [e.g. [61,89]], or by using more complex, continuous or non-grouping measures of functional diversity [90]. The selection of relevant plant traits remains, however, crucial with respect to the forest ecosystem functions to be restored. Scherer-Lorenzen *et al*. [58] provide a comprehensive list of species traits that can be used to quantify functional diversity of tree mixtures used for reforestation of European temperate forests. Selected traits included nominal (e.g. leaf type, crown architecture), ordinal (e.g. adult light requirements, height growth vigour) and scale variables (e.g. leaf N concentration, litter C:N ratio). A better mechanistic understanding of how species traits and their interactions affect ecosystem functioning is also important, however, to be able to proactively analyse different reforestation scenarios and their impact on forest functioning. In this context, it is important to realize that relationships between functional traits and ecosystem functions such as carbon storage in natural populations are not always transferable to tree plantations and vice versa [57].

### Effects of genetic diversity extend up to the ecosystem level

Whereas conservation biologists have acknowledged the negative fitness consequences of reduced genetic diversity for decades, forest restoration projects may still incorporate very few genotypes [91]. There is evidence, however, that monoclonal populations are more vulnerable to pathogens than genetically diverse assemblages [e.g. [92,93]]. The point that we want to make here, however, is that the effects of stand genetic diversity can be expected to extend far beyond the fitness of the individual trees or stands. It is only recently that it has become clear that variation in population genetic diversity or in genotype composition can have far-reaching ecological effects. The ecological consequences of genetic diversity (coined 'community genetics') have been demonstrated at different levels of organization, from the population over the community to the ecosystem [94-96]. For example, plant genotypic diversity and genotype identity have been shown to affect biomass production and community invasibility, and also the invertebrate diversity of the higher trophic levels [97,98]. It was also shown that litter decomposition and nutrient release differed between different *Populus *genotypes, indicating that selection of tree genotypes may have profound and long lasting effects on ecosystem functioning of restored forests [99,100]. Although a discipline as community genetics is in its infancy, there is already some evidence to suggest that there are extended consequences of plant genetic variation, up to the level of the ecosystem properties [96]. The selection of specific genotypes, and the genotypic diversity of tree assemblages, may therefore have major implications for the functioning and the resilience of forests [61].

### Synchronize above- and below-ground biodiversity

The above-ground biodiversity of forests also comprises fauna with important ecosystem services that include pollination, pest control and seed dispersal. The ecosystem services of birds, for instance, have been well documented [101] and in the light of forest restoration birds have been shown essential for dispersing tree seeds into restoring areas and overcoming seed dispersal and germination limitation [102,103]. Far less is known about the role of below-ground biota and the linkages between trees and these biota. The study of soil microbial community structure and functioning has traditionally received little attention in ecology. But as with above-ground biodiversity, there is evidence that below-ground diversity has a significant impact on ecosystem functioning. In a series of simplified tropical forests, Lovelock and Ewel [104] found significant positive relationships between the diversity of arbuscular mycorrhizal fungi (AMF) and ecosystem net primary productivity, and between AM fungal community evenness and ecosystem phosphorus-use efficiency. The quick development and availability of molecular tools such as t-RFLP and next-generation sequencing to quantify microbial diversity [e.g. [105,106]], together with the strong focus of the BEF approach on the functionality of ecosystems, has resulted in an increased interest in the role of microbial soil community diversity in driving processes such as organic matter decomposition and plant nutrient uptake. Because ecological restoration is usually occurring on highly disturbed or degraded sites, it is important that above-ground-below-ground species linkages are permanently considered during the restoration process [34], and more specific, that there is a synchronisation between above- and below-ground species associations [107]. Clearly, the crucial question is whether below-ground microbial community simply follows the introduced tree and shrub species, or whether some kind of inoculation is required [see e.g. [108]]. Among the relevant soil micro-organisms, arbuscular mycorrhizal fungi (AMF) and ectomycorrhizal fungi (ECMF) can be expected to play a major role during restoration of degraded sites. Many tree and shrub species associate with AMF and ECMF, which provide nutrients in exchange for plant carbohydrates. Recent evidence has shown that at least ECMF are dispersal limited, and are less abundant on isolated trees [109]. This finding may urge for some kind of active inoculation of degraded restoration sites. How to successfully apply soil microorganisms in particular restoration projects, however, is an almost empty research field. Whereas fundamental insights in the role of AMF in structuring grassland communities are growing [but see [110]], it remains largely unknown how these fungi contribute to successful restoration and the few available reports on the effects of large scale inoculations in grasslands did result in contradictory conclusions (White *et al*. [111]*vs*. Smith *et al*. [112]). Also, the inoculation of tree roots with mycorrhiza has received some attention in forest restoration projects, but the results are not straightforward [e.g., [113,114]]. This leads to the conclusion that at present much remains to be understood about how below-ground microbial diversity contributes to successful restoration of forest functions. Newly available molecular tools to quantify microbial diversity combined with detailed measurements of forest functioning are likely to increase our insights in how to apply below-ground biodiversity for restoration purposes.

### Restored forests are often novel ecosystems

While restored forests may deliver similar ecosystem services and conserve levels of biodiversity comparable to the pre-disturbance vegetation, restored forests rarely match the composition and structure of the original forest cover [115]. Large changes in ecosystems will usually result in novel systems, comprising different species, interactions and functions [116,117]. In this context, it is important to realize that both the recent tendency towards accepting perennial, global change driven changes to the environment and the increasing application of the BEF framework to ecological restoration may facilitate the acceptance of using non-native species in forest restoration. While many ecologists still consider autochthony of species a prerequisite for their use in ecological restoration [see e.g. [118]], a focus on species' functions rather than on species' origins is already advocated by others [119] as being a "more dynamic and pragmatic approach to the conservation and management of species". In this sense, the BEF approach may be at the source of a paradigm shift in restoration ecology [120].

## Conclusions

The BEF approach provides a useful framework to evaluate forest restoration in an ecosystem functioning context. It highlights different aspects of forest restoration that do not always receive sufficient attention in the more traditional approaches to restoration. At the same time the BEF framework confronts us with huge knowledge gaps still present in restoration science. The mechanistic understanding of how plant functional traits and their mutual interactions affect ecosystem functioning, understanding the role of genetic diversity in ecosystem functioning, and acquiring insights in the interactions between below-ground biodiversity and forest functioning and restoration success, are the most urgent research needs.

## Abbreviations

AMF: arbuscular mycorrhizal fungi; BEF: biodiversity - ecosystem functioning; ECMF: ectomycorrhizal fungi;NDVI: normalized difference vegetation index

## Competing interests

The authors declare that they have no competing interests.

## Authors' contributions

RA and OH contributed equally to the design of the review. The authors drafted the manuscript together. Both authors read and approved the final manuscript.

## Authors' information

Raf Aerts is a forest engineer and tropical field ecologist and is a post-doctoral research fellow at the Division Forest, Nature and Landscape of the University of Leuven (K.U.Leuven). His research focuses on (tropical) forest conservation and forest restoration. He applies principles of community ecology and ecological genetics to trees, birds, epiphytic orchids and wild arabica coffee. Olivier Honnay is associate professor of plant ecology and plant conservation biology at the Biology Department of the University of Leuven.
